# Study on the Electrical and Mechanical Properties of TiC Particle-Reinforced Copper Matrix Composites Regulated by Different Rare Earth Elements

**DOI:** 10.3390/nano15020096

**Published:** 2025-01-09

**Authors:** Denghui Li, Changfei Sun, Zhenjie Zhai, Zhe Wang, Cong Chen, Qian Lei

**Affiliations:** 1 School of Chemistry and Materials Science, Qinghai Minzu University, Xining 810007, China; li15039538994@163.com (D.L.); changfeisun@163.com (C.S.); 15562091462@163.com (Z.Z.); 13897116233@139.com (Z.W.); 2 Qinghai Key Laboratory of Nanomaterials and Technology, Xining 810007, China; 3 State Key Laboratory of Powder Metallurgy, Central South University, Changsha 410083, China

**Keywords:** powder metallurgy, composites, duplex reinforcement, rare earth modification

## Abstract

Copper matrix composites (CMCs) synergistically reinforced with rare earth oxides (Re_2_O_3_) and TiC were prepared using a powder metallurgy process with vacuum hot-pressing and sintering technology, aiming to explore new ways to optimize the properties of composites. Through this innovative approach, we propose a new solution strategy and idea for the difficult problem of mutual constraints between electrical and mechanical properties faced by traditional dual-phase reinforced Cu-matrix composites. Meanwhile, the modulation mechanism of Re_2_O_3_ in CMCs and the electrical and mechanical properties of the composites were investigated. The compressive yield strength was improved from pure Cu (50 MPa) to TiC/Cu (159 MPa). The yield strength of Eu_2_O_3_-TiC/Cu obtained after biphasic strengthening is 213 MPa, which is 326% higher than that of pure Cu, and the ultimate compressive strength reaches 790 MPa. The conductivity was enhanced from TiC/Cu (81.4% IACS) to La_2_O_3_-TiC/Cu (87.3% IACS).

## 1. Introduction

**Figure 1:**
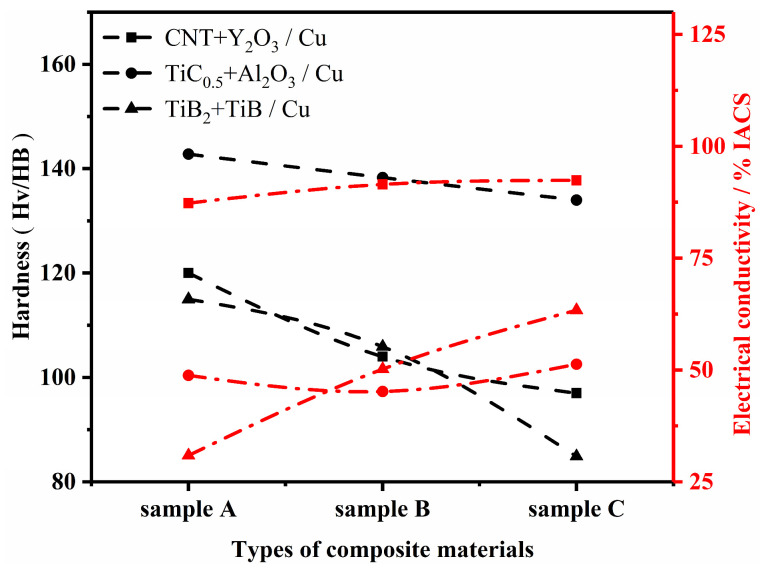
Comparative change curves of mechanical and electrical properties of conventional dual-phase reinforced CMCs literature study. (“▘” stands for the composite CNT+Y_2_O_3_/Cu, Samples A through C, respectively: 120 Hv, 87.3% IACS; 104 Hv, 91.5% IACS; 97 Hv, 92.4% IACS [18]. “●” stands for the composite TiC_0.5_+Al_2_O_3_/Cu, Samples A through C, respectively: 142.8 Hv, 48.8% IACS; 138.3 Hv, 45.2% IACS; 134 Hv, 51.3% IACS [19]. “▲” stands for the composite TiB_2_+TiB/Cu, Samples A through C, respectively: 115 HB, 30.9% IACS; 105.9 HB, 50.2% IACS; 84.9 HB, 60.4% IACS [20]).

Copper matrix composites (CMCs) are widely used in several fields due to their excellent properties [1,2,3,4,5]. The material perfectly combines the excellent electrical and thermal conductivity of copper [6,7] and the excellent mechanical properties of the reinforcing phase, exhibiting high strength, high hardness [8,9], good abrasion resistance [10,11] and corrosion resistance [12,13]. In the field of electronics industry [14], copper matrix composites are used to manufacture high-performance electronic connectors, wires and heat sinks to ensure the efficient and stable operation of electronic equipment. In the field of machinery, it is used to manufacture wear-resistant and corrosion-resistant bearings, gears and other key components to improve the service life and reliability of equipment. In addition, copper matrix composites (CMCs) play an important role in aerospace [15,16], transportation [17], and other fields, making significant contributions to the development of modern industry. With the rapid change in science and technology, the traditional single-phase reinforced CMCs can no longer meet the growing production demand, while the dual-phase reinforced CMCs are often faced with the characteristics of negative correlation between the mechanical and electrical properties of the growth, see Figure 1. Therefore, in order to meet the needs of the future development of science and technology, the optimization of the preparation process, the raw material ratios, and the development of CMCs with a more comprehensive performance has become an urgent problem to be solved at the present time.

According to different characteristics and scope of application, their preparation methods mainly include powder metallurgy [21], internal oxidation [22], hot dip coating, and discharge plasma sintering [23]. Among these methods, the powder metallurgical method is favored for its high raw material utilization, process flexibility, low pollution, and suitability for industrial production, and is widely used in the preparation of mechanical parts, functional materials, and composites. Reinforced particles are mainly classified into continuous fibers, short fibers, particles, and whisker-reinforced phases due to their morphological characteristics. Particle-reinforced phases are favored due to their easy preparation process, low cost, wide audience, simple and controllable size, and the easy diffuse distribution in the matrix. They broadly include carbides represented by TaC and TiC, borides represented by TiB_2_ and TiB, and oxides represented by TiO_2_ and Al_2_O_3_. TiC is widely considered to be the material of choice for particle-reinforced CMCs due to its high hardness, ultra-high melting point [24,25], and good chemical stability [26]. For example, M. Ravichandran et al. [27] prepared TiC/Cu composites with different contents and it was found that the composites possessed the minimum coefficient of friction when the TiC content was at 12 wt.%; Vimal K et al. [28] prepared graphite and titanium carbide biphasic reinforced copper matrix composites and the compressive strength of their composites was increased by about 108%. In addition to this, the literature has shown that Re (rare earth element) has a significant effect on enhancing the overall performance of copper matrix composites. For example, Walaa Abd-Elaziem et al. [29] prepared Y_2_O_3_/Cu-Ni composites using a powder metallurgical process, and showed that the introduction of Y_2_O_3_ can improve the hardness, yield strength, and ultimate compressive strength of the composites, but it is potentially hazardous to the physical and mechanical properties of the composites when the content is high. Jingwei L et al. [30] prepared CMCs reinforced by different levels of rare earth La-reinforced CMCs, which showed that the mechanical and electrical properties of the composites improved with increasing Re, but when the content exceeded 0.04 wt.%, the mechanical properties remained improved and the electrical properties gradually decreased. It can be concluded that rare earth elements have a significant effect on strengthening the comprehensive performance of copper matrix composites, but there is an extreme point of content, which will lead to a decline in the overall performance of the composites.

In this study, 4 wt.% TiC/Cu composites were successfully prepared by powder metallurgy process combined with vacuum hot press sintering method to explore the effect of TiC particles on the comprehensive performance of matrix materials. Combined with the fact that the optimal reinforcement content of rare earth oxides is much higher than that of pure rare earths, 1 wt.% of rare earth oxide particles, Eu_2_O_3_/La_2_O_3_/Y_2_O_3_, were introduced to investigate the changes in mechanical and electrical properties of biphasic-reinforced Cu-matrix composites under the modulation of rare earth elements. It aims to explore new ways to optimize the material properties. Through this innovative approach, we propose a new solution strategy and idea for the difficult problem faced by traditional dual-phase reinforced copper matrix composites, in which the electrical and mechanical properties are constrained by each other.

## 2. Experiments

**Figure 2:**
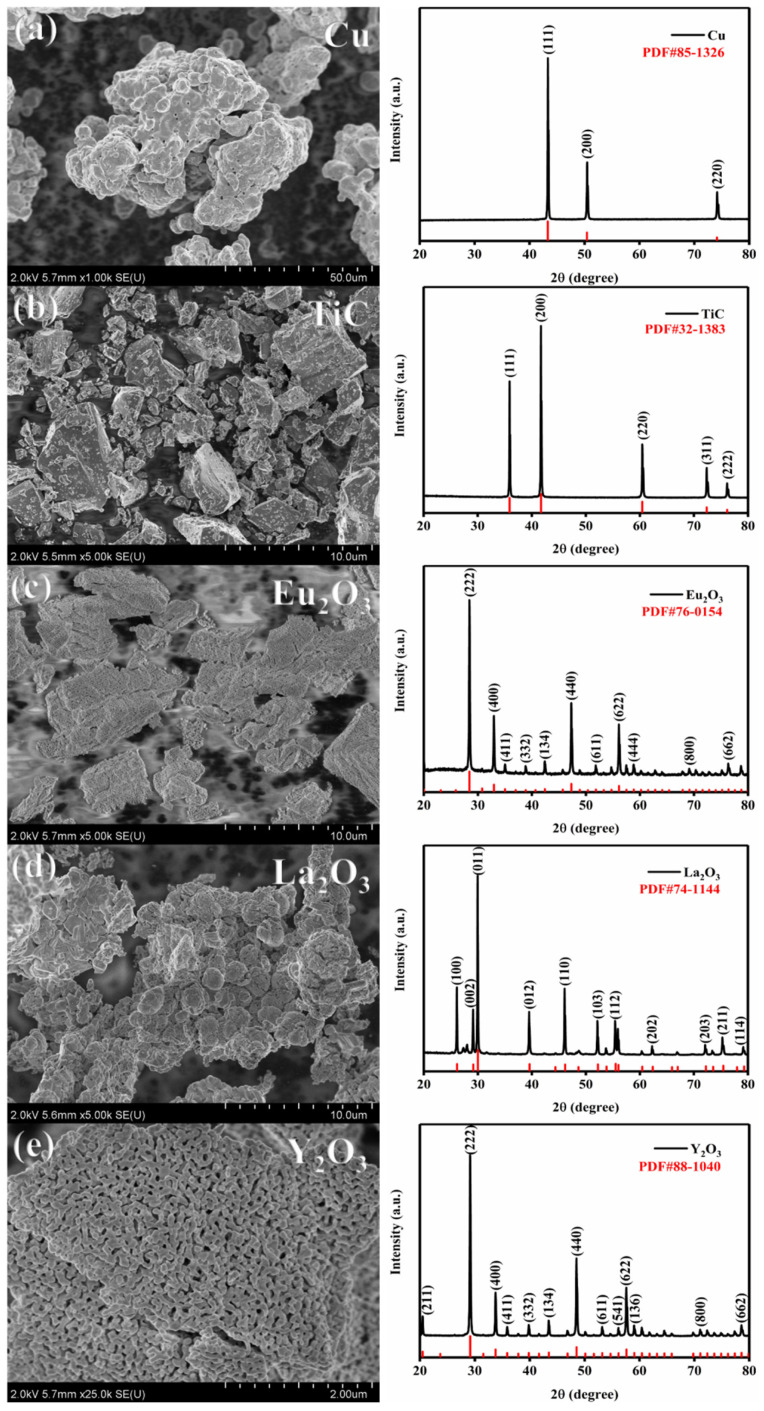
Representation of SEM and corresponding XRD images of pristine powders (**a**) Cu; (**b**) TiC; (**c**) Eu_2_O_3_; (**d**) La_2_O_3_; (**e**) Y_2_O_3_.

The raw materials used in the experiments included pure Cu powder (Approx. 45 microns), Eu_2_O_3_ powder, La_2_O_3_ powder, Y_2_O_3_ powder (1–3 microns) with a purity of 99.9%, and TiC powder (5–7 microns) with a purity of 99.99%. The experimental drugs were obtained from Shanghai, China (Shanghai Macklin Biochemical Technology Co., Ltd.). NOVA NANOSEM 230 scanning electron microscope (SEM: FEI Company, Columbia, MD, USA) and D8 ADVANCE X-ray diffractometer (XRD: Voltage 30 Kv, Current 10 mA. Scanning angles from 20° to 80° at a rate of 10°/min. Bruker Corporation, Saarbrücken, Germany) were utilized for morphological observation and elemental determination of the pristine powders, which are shown in Figure 2. From the SEM images, it is observed that the matrix powder is nearly spherical without obvious agglomeration, and the TiC and Re_2_O_3_ are irregularly shaped. The main peak positions and PDF card parameters of the pristine phase were obtained from the XRD plots, and no lattice distortion of the pristine phase was found. The collection of these test results facilitates the observation of the changes in the morphology, size and crystal structure of the matrix powder after ball milling. The ball-to-material ratio was set at 10:1, and in order to prevent agglomeration from occurring during the ball milling process, anhydrous ethanol of 99.9% purity was used as the dispersion control agent, and Sample 0 (Cu), Sample 1 (TiC/Cu), Sample 1-1 (Eu_2_O_3_-TiC/Cu), Sample 1-2 (La_2_O_3_-TiC/Cu), and Sample 1-3 (Y_2_O_3_-TiC/Cu) were successfully prepared, and the sample dosing ratio is shown in Table 1.

**Figure 3:**
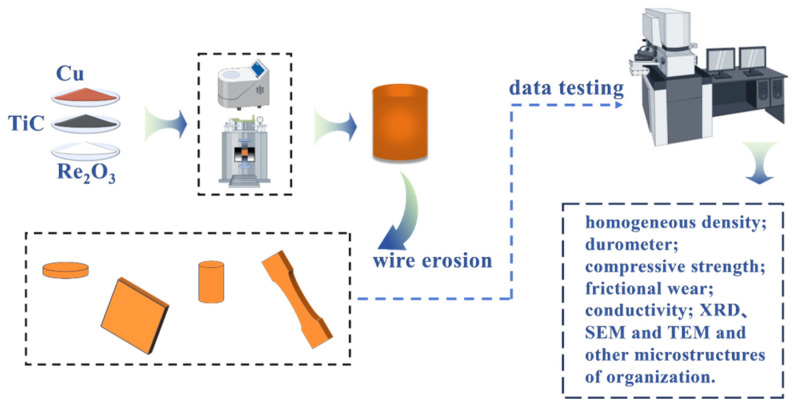
Flowchart of composite preparation and testing and analysis.

The flow of experiment and test analysis is shown in Figure 3. A QM-QX4 planetary high-energy ball mill was used, with a set rotational speed of 400 r/min and a time of 6 h. The mixed composite powders were transferred to ZT-40-20Y vacuum hot-pressing sintering furnace, with a set heating rate of 10°/min, a target temperature of 850 ℃, a load of 50 MPa, and an insulating pressure holding time of 1 h. The target specimens were obtained after sintering. The specimen was processed into standard size according to the test requirements using a wire cutter, and the surface of the specimen was polished to a flat and smooth surface with the help of sandpaper and YM-2A polishing machine. The composition of the corrosion solution: a mixture of dilute hydrochloric acid and dilute nitric acid. Finally, the friction coefficient, hardness, density, compression properties and electrical conductivity of the composites were tested and analyzed by MMW-1 friction and wear tester, HR-150A Rockwell hardness tester, ML204T/02 density analyzing balance, CMT5105 universal tester, and ST2263 vortex conductivity tester; and D8 ADVANCE X-ray diffractometer and metallurgical microscope were used, D8 ADVANCE X-ray diffractometer, metallurgical microscope, NOVA NANOSEM 230 Field Emission Scanning Electron Microscope, Tecnai G2 F30 Transmission Electron Microscope (TEM: FEI Company, Columbia, MD, USA)and other instruments to test and analyze the composite powder and material for elemental species and micro-morphology.

## 3. Results and Discussion

### 3.1. Powder and Solid Characterization Testing of Pure Cu Specimens

**Figure 4:**
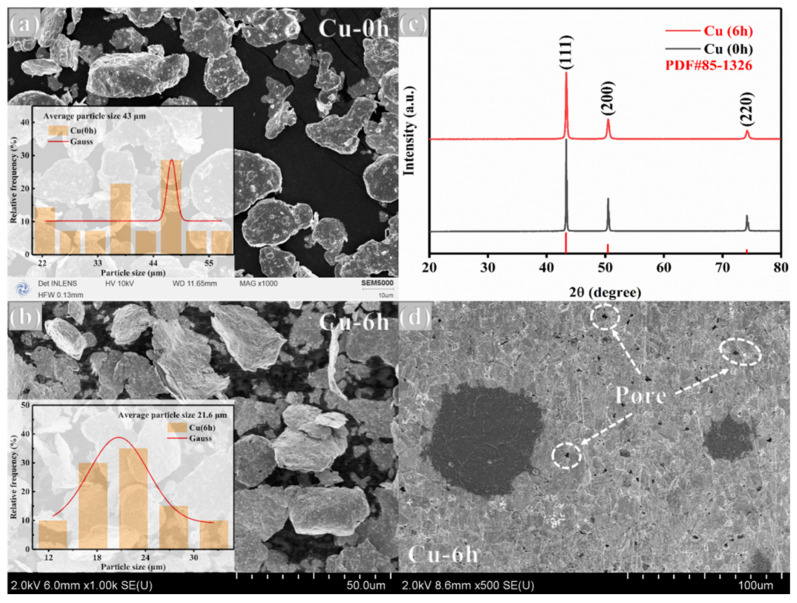
Pristine Cu characterization test set (**a**) SEM and particle size distribution of ball-milled 0 h pure Cu powder; (**b**) SEM and particle size distribution of ball-milled 6 h pure Cu powder; (**c**) XRD of ball-milled 0 h and 6 h pure Cu powder; and (**d**) SEM of pure Cu material.

Figure 4 shows the characterization test set of pristine pure Cu. From Figure 4a,b, it can be seen that the morphology of Cu particles is affected by the high-energy ball milling process, and the matrix particles are subjected to intense collision and extrusion, which transforms the original near-spherical morphology into flat flake and flat sphere morphology, and the average size is significantly reduced. Combined with the particle size distribution graph and the XRD diffraction peaks of Cu—6 h in Figure 4c, compared with those of Cu—0 h, the peak intensity is significantly reduced, and the peak broadening phenomenon obviously occurs, indicating that ball milling destroys the original crystal structure of the matrix, resulting in a decrease in the degree of crystallinity and grain refinement, which is corroborated by the intuitive results of Figure 4a,b. Observation Figure 4d reveals that the sintered material has more pores, which is due to the lower stacking efficiency [31,32] of flat lamellar and flat spherical particles compared to near-spherical particles, which is more likely to form pores during sintering.

### 3.2. Composite Powder Characterization Test for Each Specimen

**Figure 5:**
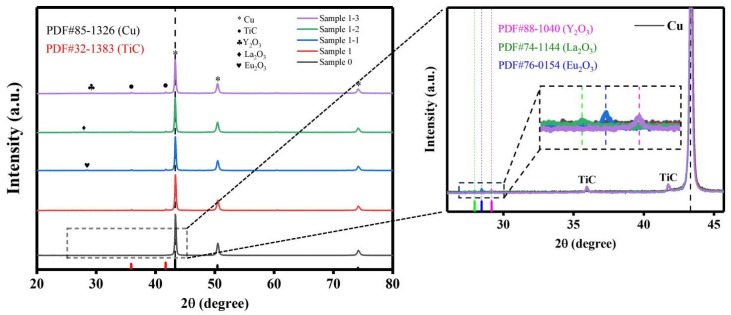
Comparison of XRD of each specimen powder after 6 h of ball milling.

Figure 5 shows the XRD comparison curves of each specimen powder after 6 h of ball milling. Referring to the PDF standard card, it can be obtained from the curve that the diffraction peaks representing Cu are 2 at 43.3°, 50.4° and 74.1°, and the diffraction peaks at 35.9° and 41.7° represent TiC. The diffraction peaks at around 28.5° belong to Eu_2_O_3_/La_2_O_3_/Y_2_O_3_, which is a weakly raised point on the XRD pattern due to the low content. After the addition of the first-phase reinforcing particles TiC, the diffraction peaks of Sample 1 show a slight decrease in peak intensity and a broadening of peaks relative to those of Sample 0, indicating that the crystallinity is still decreasing and the matrix particles are refined. After the addition of the second-phase reinforced particle Re_2_O_3_, it can be observed that the diffraction peaks of Sample 1-1, 1-2 and 1-3 have a tendency to increase in peak intensity and broaden in peak width relative to Sample 1. It is well-known that the smaller the crystal particles are the higher their crystallinity tends to be. The above image will appear: First, because most of the reinforced particles themselves have high hardness, in the high-energy ball milling process they played a role similar to the role of carbide balls, the matrix particles have a certain destruction and shearing effect. Second, the electronegativity of rare earth elements is low, easy to adsorb on the surface of the matrix particles to hinder the occurrence of agglomeration. Thirdly, the rare earth elements themselves are chemically active, and it is easy to form an interfacial reaction with the matrix phase during the ball milling process, which triggers the boundary structure instability of the matrix particles, and it is easier to achieve the purpose of destroying the surface crystal structure and refining the grains [33,34,35]. Meanwhile, comparing the XRD curves of the five specimens together, it can be found that the main peak of Cu is neither shifted nor split. This indicates that neither solid solution reaction nor new phase appears in the process of high-energy ball milling, and the stability of the crystal structure is better.

**Figure 6:**
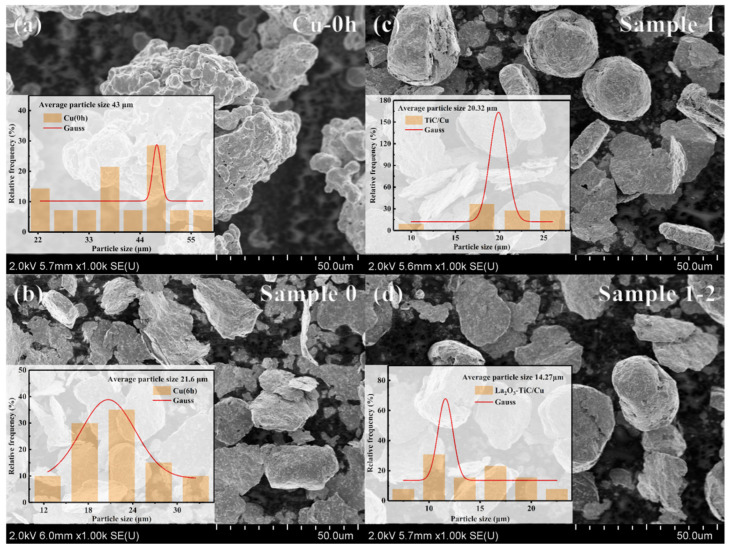
SEM and particle size distribution images of some specimen powders (**a**) ball-milled 0 h pure Cu powder; (**b**) ball-milled 6 h pure Cu powder; (**c**) ball-milled 6 h TiC/Cu composite powder; and (**d**) ball-milled 6 h TiC-La_2_O_3_/Cu composite powder.

Figure 6 shows the SEM and particle size distribution images of some specimen powders. It can be concluded that the matrix particle size after high-energy ball milling is reduced from 43 microns to 21.6 microns for pure copper, and the particle shape of the matrix phase in the composite powder with the presence of the reinforcing phase is more likely to maintain a near-spherical morphology, and the grain size and distribution tend to be more diffuse. Taking La_2_O_3_ as an example, after adding the second phase rare earth oxides [36,37] to strengthen the particles, the matrix grain size is reduced to 14.27 microns, with an obvious fine-grained reinforcement [38,39,40] effect; this can be seen in the ball milling stage, where Re_2_O_3_ has played the role of grain refining.

**Figure 7:**
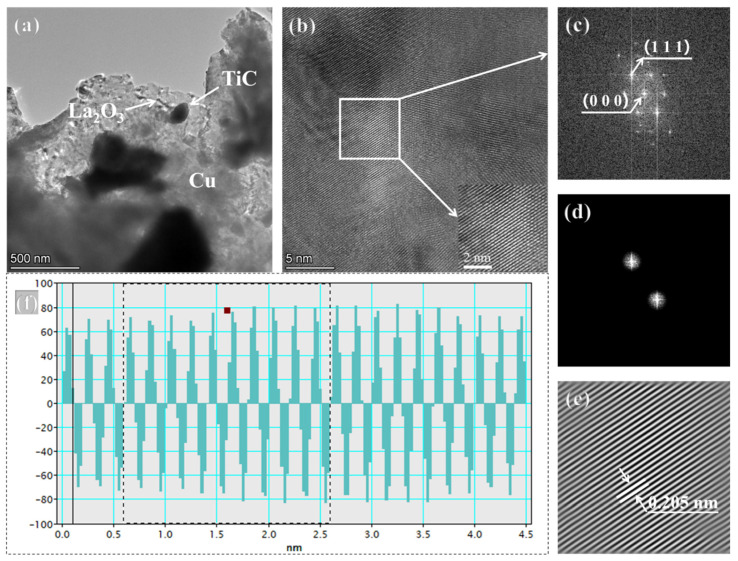
Sample 1-2 composite powder TEM and processing analysis set (**a**) Sample 1-2 powder TEM; (**b**) Sample 1-2 powder high-resolution TEM; (**c**) diffractogram; (**d**) symmetric diffraction spot; (**e**) lattice fringes; (**f**) crystal spacing.

Figure 7 shows the TEM of Sample 1-2 composite powder and the group of processing analysis. Figure 7a A high-resolution TEM of the Sample 1-2 composite powder, which can be represented by bright gray continuous regions for Cu and black blocky regions belonging to TiC. Due to the larger relative mass and smaller particle size of La_2_O_3_, it appears as small dark black spots on the TEM image. Figure 7b White box lines were used to identify the selected diffraction regions in the high-resolution TEM image, with local magnification in the lower right corner. The presence of a partially amorphous condition is also observed in the upper right corner of the figure, which is the result of ball milling leading to sufficient energy gain in the composite powder, which in turn transforms to the amorphous state [41,42]. The Fourier transform of the selected area yields the diffractogram Figure 7c, and the masking yields the diffraction spot map with a spacing of 2d Figure 7d. The inverse Fourier transform gives the lattice fringes Figure 7e, and the measurement of crystal spacing on Figure 7f gives d_Cu(111)_ = 0.205 nm. Comparison with the standard PDF card data of d_Cu(111)_ = 0.208 nm reveals that there is no significant change in d, which indicates that the grain refinement is carried out by physical means such as collision and extrusion, destructive shear, etc., and that the crystal structure is still intact without any distortion.

### 3.3. Composite Material Characterization Test for Each Specimen

**Figure 8:**
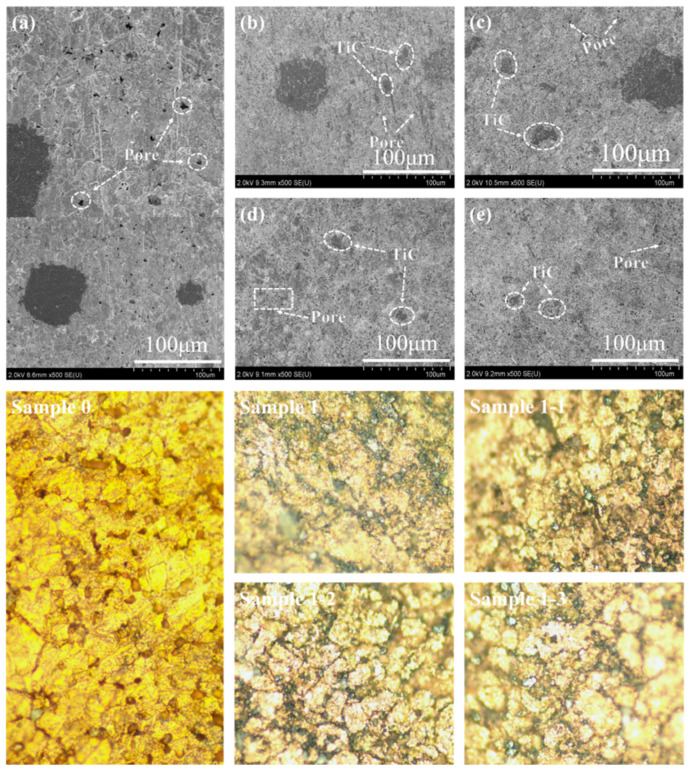
SEM and metallographic specimen images of each specimen material (**a**) Sample 0; (**b**) Sample 1; (**c**) Sample 1-1; (**d**) Sample 1-2; (**e**) Sample 1-3.

Figure 8 shows the SEM and metallographic specimens of each sample composite. From the SEM and metallography of (a) Sample 0, it can be observed that it has good interfacial bonding, but the distribution of pores is more, and the pore size is larger. This is because the ball milling process changes the matrix grain morphology from near-spherical to flat-spherical and flat-flaky, the stacking efficiency decreases, and a large number of pores appears after sintering. After the introduction of TiC particles, the number of pores and the size of the pore diameter in (b) decreased significantly, but there was agglomeration in the reinforced phase [43]. This is due to the fact that TiC particles are uniformly distributed in the matrix, which can act as heterogeneous nucleation points to promote the exclusion of melt gases during sintering and fill the pores under the applied load, reducing the generation of pores. The number of pores in (c–e) is higher relative to Sample 1, and the pore diameter is also slightly larger. This is due to the fact that the rare earth oxides are prone to reacting with other impurities in the copper matrix to form high melting point compounds, which are unevenly distributed in the molten state of the composite copper liquid, affecting the melting infiltration and solidification process of the composites, and increasing the porosity. Comparatively, it can be observed that Sample 1-1 has the least number of porosities and the smallest pore size, Sample 1-3 is next to it and Sample 1-2 performs the worst. The visualization of the metallographic specimen maps of each sample is consistent with SEM and can be corroborated.

**Figure 9:**
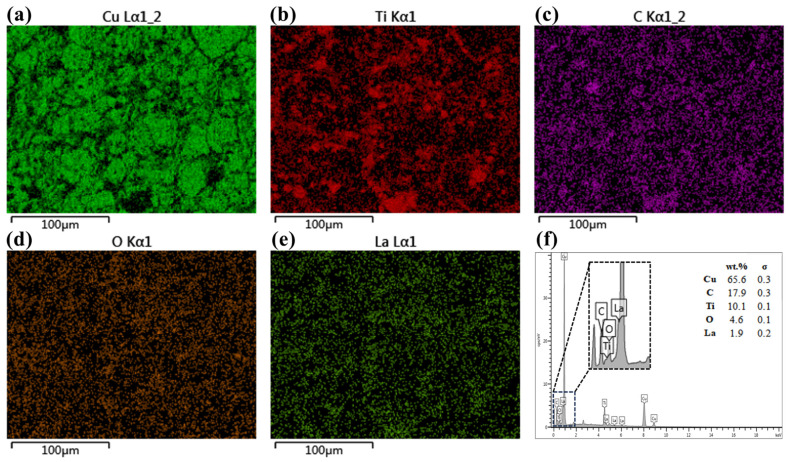
Sample 1-2 composites EDS and mapping (**a**) Cu; (**b**) Ti; (**c**) C; (**d**) O; (**e**) La; (**f**) EDS energy spectra.

Figure 9 shows the EDS spectra of Sample 1-2 composites with mapping. Green represents the Cu matrix, red represents Ti, purple represents C, brown represents O, and dark green represents La. Observation of the spectra reveals that the distribution of TiC and La_2_O_3_ after sintering is diffuse, but there is a small part of the TiC bias phenomenon in accordance with the SEM results of the powder in Figure 8d. The elemental species of the Sample 1-2 composites can also be qualitatively and quantitatively analyzed by EDS energy spectroscopy.

### 3.4. Mechanical Properties of Composite Materials for Each Specimen

**Figure 10:**
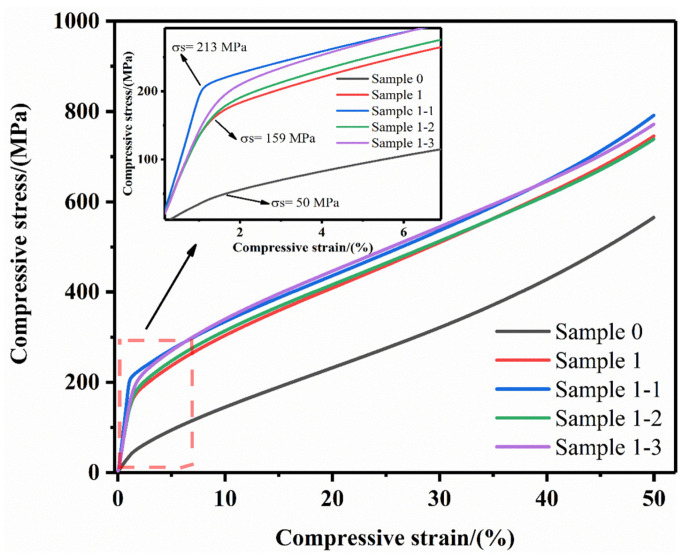
Comparison curve of compressive strength of each specimen material.

Figure 10 shows the comparative compressive curves of Sample 0, Sample 1, Sample 1-1, Sample 1-2 and Sample 1-3 specimens. It is observed that the compressive strains of each sample are larger, which is due to the excellent ductility of the Cu matrix and the low content of the reinforcing phase, so the material’s deformation ability is good. The yield strength of Sample 0 is low, which is only 50 MPa, and it is increased to 159 MPa after the introduction of the reinforcing phase TiC, and then it is increased twice by the introduction of Re_2_O_3_. This is due to the soft texture of the matrix Cu, and the Orowan mechanism of diffusion reinforcement [44,45] works after the introduction of TiC, which effectively prevents the movement and accumulation of dislocations and improves the strength and stability of the composites. Combined with the purifying and de-hybridizing effects of Re_2_O_3_ and the strengthening of grain boundaries, the yield strength and stability of the composites are enhanced twice. Observing the yield point details of the enlarged red dashed line, it can be seen that the yield strengths of the composites of Sample 1-1, 1-2, and 1-3 are increased to 213 MPa, 167 MPa, and 185 MPa, respectively, relative to that of Sample 1, Sample 1-1 has the best improvement effect, and the ultimate compressive strength can reach 790 MPa.

**Figure 11:**
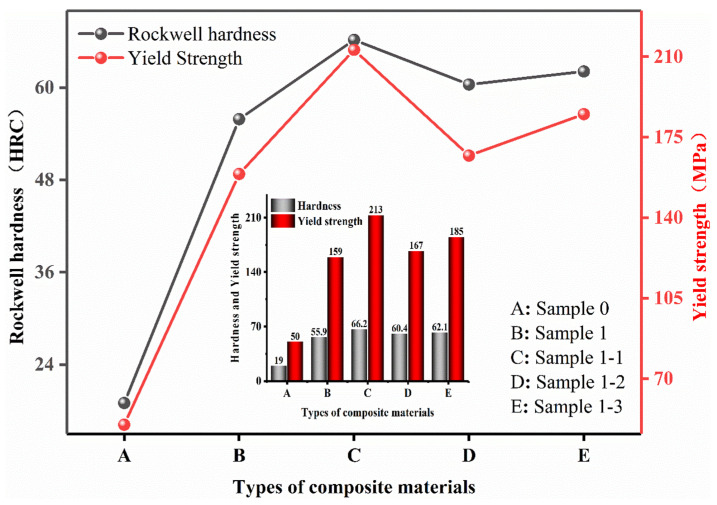
Hardness–yield point comparison curves for each specimen material.

**Figure 12:**
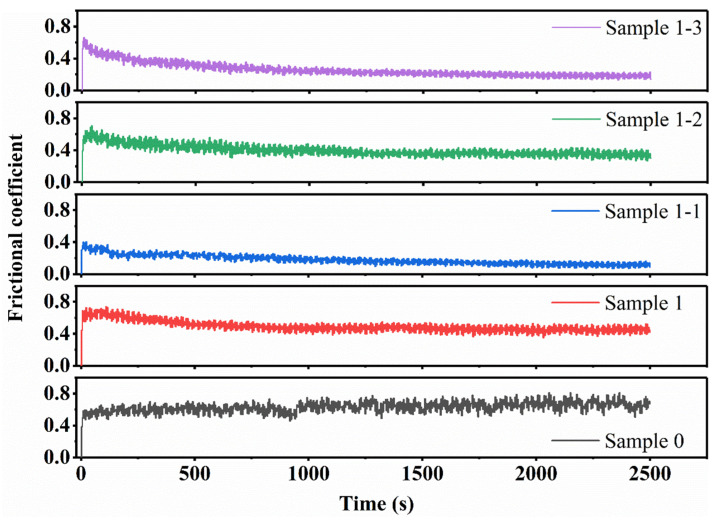
Comparison curve of friction coefficient of each specimen material.

**Figure 13:**
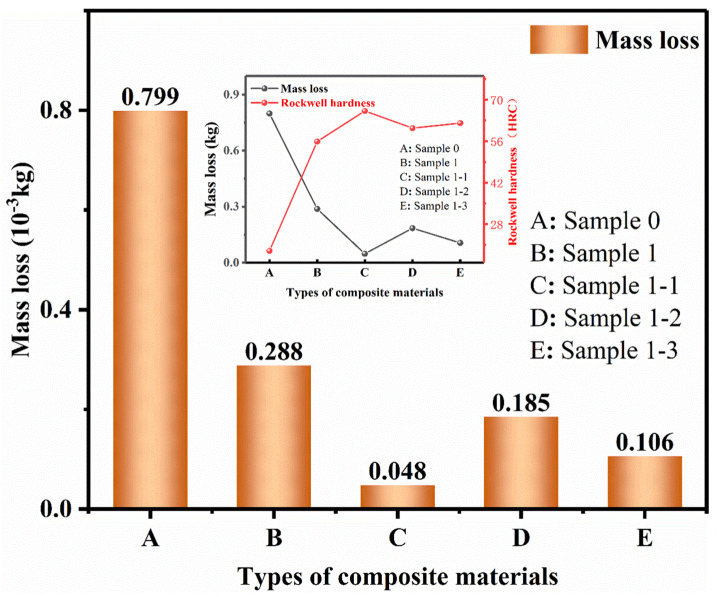
Wear volume–hardness comparison curves for each specimen material.

The red curve in Figure 11 represents the change in the yield strength of each specimen and the gray curve represents the change in the Rockwell hardness (HRC) of each specimen. The study shows that the hardness of the pure Cu material is low, the addition of TiC has significantly increased the hardness value, and the continued addition of Re_2_O_3_ shows a secondary increase in the hardness change. Combined with Figure 4 this is because the pure Cu material texture is soft, high-energy ball milling resulting in particle morphology from the near-spherical to oblate spherical, oblate flaky transformation, the stacking efficiency decreases, the material porosity increases after sintering, the structure of the loose, easy to slip deformation under the action of the applied load. The reinforcing phase TiC played a role in diffusion reinforcement after addition, effectively hindering the movement and stacking of dislocations, and the strength and mechanical properties of the composites were improved. Continuing to add rare earth oxides, combined with Figure 6, Figure 8 SEM is its role in grain refinement, strengthening the role of grain boundaries, the strength and mechanical properties of the composite material has been enhanced for the second time. Observing the hyperbolic trend and comparing the histogram intuitive data, it can be found that the hardness change in the composite material of each sample is consistent with the change in compressive yield strength, which is consistent with the existing public mechanics research. Figure 12 shows the comparative curves of friction wear variation for Sample 0, Sample 1, Sample 1-1, Sample 1-2, and Sample 1-3. It is observed from the figure that the friction coefficients of all curves show large fluctuations in the initial operation, which is due to the uneven force of the applied load, the specimens are in the stage of adhesive wear [46,47,48], and the fluctuations in the curves are gradually stabilized when the friction layer is formed. As the friction time continues to increase, the average friction coefficient curve stabilizes when the friction time reaches more than 1500 s. The average coefficient of friction decreases from 0.65 for Sample 0 to 0.5 for Sample 1 and continues to decrease to within 0.2 with the addition of Re_2_O_3_. It can also be observed from Figure 13 that the wear amount of each sample material is negatively correlated with its hardness change. Combined with Figure 8 metallography, it can be seen that the pure Cu material has more pores and a loose structure, resulting in a high coefficient of friction. The addition of diffusely reinforced TiC particles plays a role in making the composite material wear resistance increase, as well as continuing to add Re_2_O_3_ second-phase reinforcement, refine the grain, strengthen the grain boundaries, and fill the pores under the action of the applied load during sintering, which makes the composite material surface smoother and better stability.

### 3.5. Density and Electrical Properties of Each Specimen Material Test

**Figure 14:**
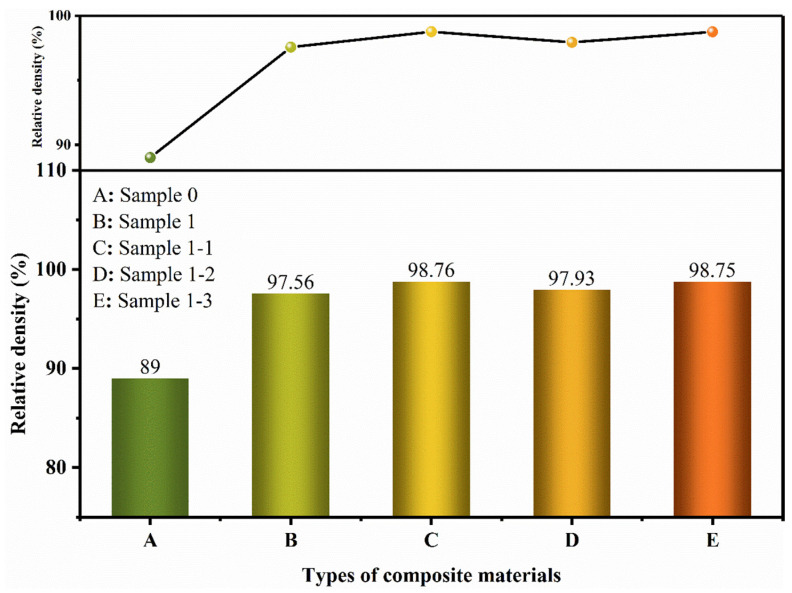
Comparison of material densities for each specimen.

Figure 14 shows the density comparison images of Sample 0, Sample 1, Sample 1-1, Sample 1-2, and Sample 1-3, and the density of Sample 0 is only 89%, which is due to the extrusion, collision, and deformation by mechanical stress, and the powder accumulation is changed from the near-spherical shape with high accumulation efficiency to the flat spherical and flat flake shape with low accumulation efficiency. After sintering, more pores with larger pore size appear, which affects the densification. Combined with Figure 6 and Figure 8, it is found that by adding the reinforcing phase TiC, the reinforcing phase moves and fills the pores under the applied load, and the density of the composites increases to 97.56%. A secondary increase in density occurs with the addition of the second-phase Re_2_O_3_. This is due to the effect of rare earth elements in refining the grain and purifying the impurities to reduce the pores and defects caused by the grain size or impurities. At the same time, the effect of strengthening the grain boundary can also improve the densification by improving the interfacial bonding between the reinforcing phase and the Cu matrix.

**Figure 15:**
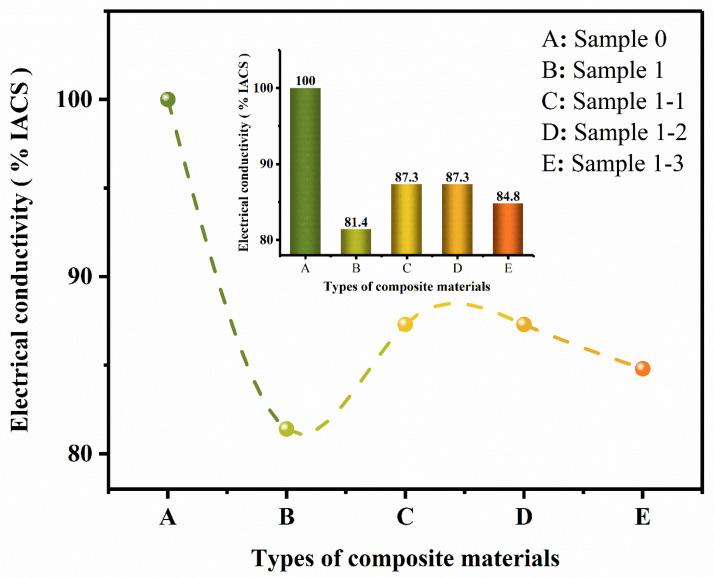
Comparison of electrical conductivity of each specimen material.

Figure 15 shows the conductivity comparison curves for Sample 0, Sample 1, Sample 1-1, Sample 1-2, and Sample 1-3. Observing the histogram pure Cu data conductivity is not ideal; in conjunction with Figure 4, this is due to the fact that the material is more porous, increasing the probability of electron scattering and the conductivity decreases. With the addition of enhanced phase TiC, conductivity further decreased; this is because the conductivity of TiC and copper conductivity are more different, leading to a decrease in conductivity. Continuing the addition of the second-phase Re_2_O_3_ the electrical conductivity rebounded significantly, which is consistent with the findings of the literature [29,30]. This is because the rare earth oxides refine the grain, purification and removal of impurities, improve the role of grain boundaries to reduce the probability of the composite material pores and defects, reduce the probability of electron scattering, and enhance the electrical conductivity of the composite material.

## 4. Conclusions

In this paper, five samples, including Sample 0, Sample 1, Sample 1-1, Sample 1-2, and Sample 1-3, were successfully prepared by wet grinding according to the ball material ratio of 10:1 and using the powder metallurgy process combined with the vacuum hot-pressing sintering method. After microscopic characterization of the specimen powders and solids as well as physical, electrical, and mechanical property tests of the solid samples, the following conclusions were drawn:

(1) The high-energy ball milling process on pure Cu powder, TiC/Cu composite powder and Re_2_O_3_-TiC/Cu composite powder has produced the effect of grain refinement; compared with Figure 6, this shows that the second-phase Re_2_O_3_ also has the effect of grain refinement.

(2) By the mechanical external force of extrusion, collision, deformation, Cu particles of the original high stacking efficiency of the near-spherical morphology into a low stacking efficiency of the flat spherical, flat flake morphology, porosity enhancement, the material densification, hardness, wear resistance is low. The addition of TiC and the second-phase Re_2_O_3_ composites have an obvious relative enhancement effect on density, hardness, and wear resistance.

(3) With the addition of TiC, the compressive yield strength of the composites was increased from 50 MPa to 159 MPa for pure Cu, and the addition of the second phase, Re_2_O_3_, continued to increase the compressive yield strength to 213 MPa, 167 MPa, and 185 MPa. The Sample 1-1 (Eu_2_O_3_-TiC/Cu) specimen, which has the strongest mechanical properties, shows an increase in yield strength of 326% with respect to pure Cu and 34% with respect to Sample 1 (TiC/Cu), with a maximum compressive strength of up to 790 MPa.

(4) After the addition of TiC, the electrical conductivity of Sample 1 (TiC/Cu) specimen decreased to 81.4% IACS, and after we continued to add the second-phase Re_2_O_3_, the electrical conductivity rebounded to 87.3% IACS, 87.3% IACS, and 84.8% IACS, respectively. The electrical properties of Sample 1-1 (Eu_2_O_3_-TiC/Cu), Sample 1-2 (La_2_O_3_-TiC/Cu) composites showed the best electrical properties.

In summary, Sample 1-1 (Eu_2_O_3_-TiC/Cu) composites have the best overall performance. First, the fine crystalline reinforcement brought by the ball milling process. Secondly, the reinforcing phase TiC is diffusely distributed in the matrix phase where the Orowan mechanism plays a role, which effectively hinders the movement and accumulation of dislocations and enhances the mechanical properties of the composites. Third, the addition of the second-phase Re_2_O_3_ refines the grains, strengthens the grain boundaries, purifies and removes impurities to reduce the lattice distortion and the probability of electron scattering, improves the electrical conductivity, and thus enhances the comprehensive performance of the composites.

## Figures and Tables

**Table 1:** Specimen dosing ratios.

Sample Number	Cu	TiC	Eu_2_O_3_	La_2_O_3_	Y_2_O_3_
Sample 0	100 wt.%	/	/	/	/
Sample 1	96 wt.%	4 wt.%	/	/	/
Sample 1-1	96 wt.%	4 wt.%	1 wt.%	/	/
Sample 1-2	96 wt.%	4 wt.%	/	1 wt.%	
Sample 1-3	96 wt.%	4 wt.%	/	/	1 wt.%

## Data Availability

Data will be made available on request.
