# Correction: Li et al. Study on the Electrical and Mechanical Properties of TiC Particle-Reinforced Copper Matrix Composites Regulated by Different Rare Earth Elements. *Nanomaterials* 2025, *15*, 96

**DOI:** 10.3390/nano15221698

**Published:** 2025-11-10

**Authors:** Denghui Li, Changfei Sun, Zhenjie Zhai, Zhe Wang, Cong Chen, Qian Lei

**Affiliations:** 1 School of Chemistry and Materials Science, Qinghai Minzu University, Xining 810007, China; li15039538994@163.com (D.L.); changfeisun@163.com (C.S.); 15562091462@163.com (Z.Z.); 13897116233@139.com (Z.W.); 2 Qinghai Key Laboratory of Nanomaterials and Technology, Xining 810007, China; 3 State Key Laboratory of Powder Metallurgy, Central South University, Changsha 410083, China

Prior to the publication of the original work [1], reference 27 was retracted. Concerns were raised about this matter, so the following reference was removed from the reference list:

“27. Mohanavel, V.; Ravichandran, M.; Ali, K.S.A.; Sathish, T.; Karthick, A.; Arungalai, V.S.; Velmurugan, P.; Salmen, S.H.; Alfarraj, S.; Sivakumar, S.; et al. Synthesis and Workability Behavior of Cu-X wt.% TiC (x = 0, 4, 8, and 12) Powder Metallurgy Composites. *Bioinorg. Chem. Appl.* **2022**, *2022*, 8101680.”

Additionally, the sentence originally citing reference 27 was deleted:

“V. Mohanavel et al. [27] prepared CMCs reinforced with different TiC contents by powder metallurgy method and the results showed that the density and hardness of the composites increased gradually with the increase in TiC content”.

Following the removal of reference 27, subsequent references and their corresponding citations in the main text have been adjusted according to numerical sequence.

The authors state that the scientific conclusions are unaffected. This correction was approved by the Academic Editor. The original publication has also been updated.
